# Screening for SARS-CoV-2 and Other Coronaviruses in Urban Pigeons (Columbiformes) from the North of Spain under a ‘One Health’ Perspective

**DOI:** 10.3390/microorganisms12061143

**Published:** 2024-06-04

**Authors:** Aránzazu Portillo, Cristina Cervera-Acedo, Ana M. Palomar, Ignacio Ruiz-Arrondo, Paula Santibáñez, Sonia Santibáñez, José A. Oteo

**Affiliations:** 1 Centro de Rickettsiosis y Enfermedades Transmitidas por Artrópodos Vectores (CRETAV), Departamento de Enfermedades Infecciosas, Hospital Universitario San Pedro-Centro de Investigación Biomédica de La Rioja (HUSP-CIBIR), C/Piqueras, 98-3ª Planta, 26006 Logroño, La Rioja, Spain; ccervera@riojasalud.es (C.C.-A.); irarrondo@riojasalud.es (I.R.-A.); psantibanez@riojasalud.es (P.S.); ssantibanez@riojasalud.es (S.S.); 2 Departamento de Patología Animal, Facultad de Veterinaria, Instituto Universitario de Investigación Mixto Agroalimentario de Aragón (IA2), Universidad de Zaragoza, 50013 Zaragoza, Aragón, Spain

**Keywords:** avian coronaviruses, COVID-19, ‘One Health’, pigeons, RNA-dependent RNA polymerase (*RdRp*), SARS-CoV-2

## Abstract

Coronaviruses have a major impact on human and animal health. The SARS-CoV-2, a beta coronavirus responsible for the COVID-19 pandemic, is a clear example. It continues circulating and causes human deaths, and its high replication rate results in numerous variants. Coronaviruses adapt to birds and mammals and constitute a serious threat, and new viruses are likely to emerge. Urban pigeons (Columbiformes) are synanthropic birds of great interest from a ‘One Health’ perspective, due to their interaction with humans and other animals. Aware that they may act as viral reservoirs and contribute to their spread, we aimed to investigate the possible presence of SARS-CoV-2 and other coronaviruses in Columbiformes in the city of Logroño, Spain. Oropharyngeal and cloacal swabs were tested using real-time (N1 and E genes from SARS-CoV-2) and conventional PCR assays (*RdRp* gene from all coronaviruses). SARS-CoV-2 was not detected. A total of 13.3% of pigeons harbored coronaviruses closely related to Gamma coronavirus (Igacovirus) from Columbiformes in Finland, Poland and China. Monitoring the emergence of a new variant of SARS-CoV-2 capable of infecting Columbiformes should continue. SARS-CoV-2 is still circulating, the viral RNA of this virus has been detected in avian species (*Phasianidae* and *Anatidae*), and other coronaviruses are associated with animals that are in close contact with humans. The presence of Gamma coronavirus in urban pigeons must be considered for the risk of surveillance of human infections.

## 1. Introduction

Coronaviruses (CoV) (family *Coronaviridae*, subfamily *Orthocoronavirinae*) have major impact on human and animal health [1,2,3,4]. According to the International Committee on Taxonomy of Viruses [5], and based on phylogeny and genetic structures, members within this subfamily belong to four genera: *Alpha*-, *Beta*-, *Gamma*- and *Delta*-CoV. They are viruses adapted to different species of birds and mammals. Birds usually harbor *Gamma*- and *Delta*-CoV, whereas *Alpha*- and *Beta*-CoV are known to affect humans [1]. A clear example is SARS-CoV-2, a *Beta*-CoV responsible for the COVID-19 pandemic, which still circulates and causes human deaths, and whose high replication rate causes numerous variants that emerge every few months [3,6,7,8].

Viruses of the SARS-CoV group constitute a serious threat not only because of their ability to accumulate mutations but also due to the possibility of the emergence of new viruses through recombination events at a very high frequency [9]. Since animals (bats, civets, camelids, rodents, etc.) are the natural reservoirs or intermediate hosts of all CoV that affect humans [10], the detection and identification of animal CoV is a global challenge that can serve as a first step to assess their zoonotic potential. So far, the range of hosts of SARS-CoV-2 is mainly limited to humans and various mammals. Nevertheless, avian species naturally infected (RNA detection) by SARS-CoV-2 have been occasionally reported [11].

Urban pigeons (Columbiformes) are synanthropic birds of great interest under a ‘One Health’ approach due to their interaction with humans and other animals (domestic, wild and intended for food production). Aware that pigeons can act as reservoirs of viruses (including CoV) and contribute to their dissemination, and considering the demonstrated spillover potential of SARS-CoV-2 from one animal species to another [11,12], the objective of this study was to investigate the presence of SARS-CoV-2 and other CoV that could be circulating among pigeons in the city of Logroño, La Rioja, the north of Spain.

## 2. Materials and Methods

This study was approved by the Ethics Committee on Animal Experimentation from the Center for Biomedical Research of La Rioja (CIBIR) (IRA-02), and conducted according to animal welfare guidelines from the World Organization for Animal Health [13]. Columbiformes’ samples were collected by a veterinarian, with permission given by animal protection technicians from Logroño City Council and ADDA OPS S.A. (Spain).

During August/September 2021 and January/February 2022, oropharyngeal and cloacal swab specimens from 203 Columbiformes (202 *Columba livia* and one *Streptopelia decaocto*) captured at different urban locations as part of the pigeon population control in the city of Logroño, were individually collected. Samples were preserved in tubes containing Dulbecco’s Modified Eagle Medium with penicillin (100 units/mL) and streptomycin (100 μg/mL) (Sigma-Aldrich, Manheim, Germany) in field refrigerators at 4 °C and transported to the Special Pathogens Laboratory-Center of Rickettsiosis and Arthropod-Borne Diseases (CRETAV), San Pedro University Hospital-CIBIR in Logroño (the north of Spain). Specimens were split into aliquots in a class II biosafety cabinet at a biosafety level-2 (BSL-2) facility and kept at −80 °C.

Total RNA was manually extracted from 200 µL aliquots using an RNeasy Mini kit (Qiagen, Hilden, Germany), including DNA digestion with the RNase-free DNase Set (Qiagen, Hilden, Germany). RNA extracts were eluted in 65 µL of RNase-free water and stored at −80 °C before use. Reverse transcription to cDNA was carried out using an Omniscript RT commercial kit (Qiagen, Hilden, Germany).

Quantitative reverse transcription polymerase chain reaction (RT-qPCR) of a conserved endogenous beta-actin fragment gene was performed as an internal control in all samples to evaluate the reliability of the sampling and/or extraction process [14]. Potential evidence of SARS-CoV-2 was investigated using a specific one-step RT-qPCR assay targeting the partial nucleocapsid protein gene (N1) [15]. Subsequently, 10% of randomly chosen samples were screened for the SARS-CoV-2 envelope protein-encoding gene (E) [16]. Assays were conducted under previously described conditions [17]. Synthetic plasmids containing the complete N gene (Integrated DNA Technologies, Leuven, Belgium) and E gene (Eurofins Genomics, Ebersberg, Germany) were used as positive controls for SARS-CoV-2 detection, and distilled water as negative control. Samples and controls were tested in triplicate.

In addition, oropharyngeal and cloacal RNA from the same pigeon was pooled before reverse transcription, and cDNA samples were used as templates for two panCoV conventional polymerase chain reaction (PCR) (simple and nested one), targeting fragments of a conserved region within the RNA-dependent RNA polymerase (*RdRp*) gene using degenerated primers and according to the previously reported methodology [18,19]. Positive (a synthetic plasmid containing partial porcine epidemic diarrhea virus, KF468752) and negative controls (distilled water) were included in all assays.

Amplicons of the expected size were identified by agarose gel electrophoresis and sequenced in both senses. Nucleotide sequences were compared with those available in GenBank using the Basic Local Alignment Search Tool (BLAST) search [20].

Phylogenetic analysis was based on 37 partial alignments of 464-nucleotide *RdRp* sequences from pigeon *Gamma*-CoV. The evolutionary history was inferred by using the Maximum Likelihood method, the General Time-reversible model, a discrete Gamma distribution and a proportion of invariable sites (GTR + G + I), with nucleotide substitution selected according to the Akaike information criterion implemented in MEGA11 [21]. The *RdRp Igacovirus* KJ690955 from a cloacal swab of a bean goose (*Anser fabalis*) was included as an outlier.

New viral *RdRp* gene sequences of high quality generated in this study (n = 26) were deposited in GenBank under accession numbers OQ957548-56. One inconclusive sequence showing many ambiguous positions is available upon request to authors.

## 3. Results

The internal (beta-actin gene) positive and negative controls worked properly in all cases. SARS-CoV-2 virus RNA was not detected in either oropharyngeal or cloacal swab specimens when using RT-qPCR assays targeting the N gene (as first screening) and E gene (as confirmatory test). Conversely, the genetic material of other CoV was found in swabs corresponding to 27 *C. livia* out of the 203 studied Columbiformes (13.3%) when PCR assays targeting the *RdRp* gene of all coronavirus lineages were used. Sensitivity was higher using nested than one-round PCRs (13.3% vs. 2%).

**Figure 1:**
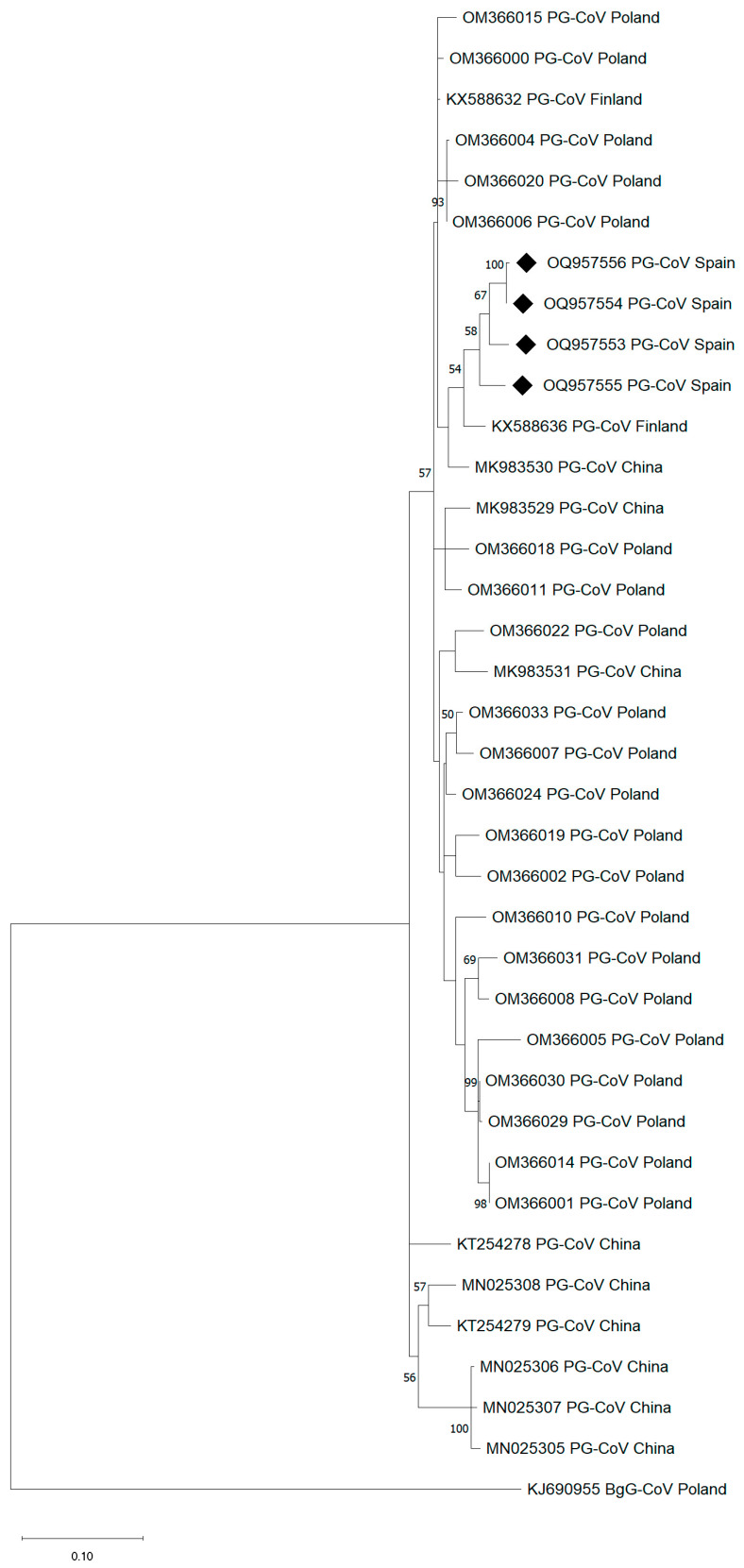
Phylogenetic analysis based on 464-nucleotide RNA-dependent RNA polymerase (*RdRp*) sequences from *Gamma* coronaviruses. Sequences from pigeons in Spain detected in this study are marked with ◆. The tree is drawn to scale, with branch lengths measured in the number of substitutions per site. Numbers (>50%) shown at the nodes correspond to bootstrapped percentages (for 1000 repetitions). PG-CoV, pigeon *Gamma* coronavirus; BgG-Cov, bean goose *Gamma* coronavirus. KJ690955: outlier.

Partial *RdRp* nucleotide sequences obtained from 26/27 pigeons clustered together and shared ≥96.4% similarity with each other. They showed the highest identity (96.1–97.2%) with sequences of avian CoV belonging to the genus *Gamma*-CoV, subgenus *Igacovirus*, found in Columbiformes, mainly in Finland, Poland and China [22,23,24] (Figure 1). When translated into proteins, *RdRp* amplicons from this study had <2.1% heterogeneity, showing the highest identity (98.9–100%) with protein sequences found in Columbiformes from Poland and China.

## 4. Discussion

From the beginning of the COVID-19 pandemic, SARS-CoV-2 has been detected in a great variety of wildlife and domestic animals, either because of natural infection or after contact with humans, as we first demonstrated in Europe with the finding of an infected cat that lived with a COVID-19 patient during the lockdown period in Spain [17]. Focusing on birds, these did not seem to be susceptible to SARS-CoV-2 in 2020, according to the first evidence of experiments performed with chickens and ducks [25,26]. However, the first findings of viral RNA detection in avian species were reported in 2021/2022 [11]. As SARS-CoV-2 becomes endemic and evolves, the ability of emerging variants to jump the interspecies barrier increases, and recent structure simulation models evidence that certain SARS-CoV-2 variants could infect chickens [27]. In addition, other genera of CoV are known to circulate in fowls and wild bird species such as chickens, turkeys, sparrows, mallard ducks, feral pigeons and graylag geese, among others [4,22,28]. Cross-infections between chickens and pigeons are considered plausible since almost identical viral sequences were found when individuals of both species were examined [29]. If coinfection of SARS-CoV-2 and a CoV from a different genus occurred in the same cell from the same host, a recombinant virus could emerge with possible ‘One Health’ devastating consequences. Aware of the impact of these global threads, we herein investigated the potential concomitant circulation of SARS-CoV-2 and other CoV in urban pigeons in the city of Logroño (Spain). Whereas SARS-CoV-2 was not detected in any of the 203 analyzed Columbiformes, the exposure to CoV in pigeons was documented for the first time in Spain using molecular tools. In our study, *RdRp* was the target gene for CoV PCRs, thus revealing that Columbiformes in the north of Spain harbored *Gamma*-CoV closely related to those found in *Columba* spp. from Northern Europe and Asia (Figure 1) [22,23,24]. Other previous studies of CoV screening undertaken with pigeons in Norway and Brazil detected *Gamma*-CoV using the nucleocapsid or the spike genes [28,30], and isolation and partial characterization of CoV-causing pancreatitis in pigeons was achieved in China [29]. In the present study, sampling for viral surveillance in pigeons was carried out in two time slots (two months each) when the COVID-19 incidence in Spain was high. Nevertheless, a longitudinal study to assess the potential circulation of CoV in pigeons over time would be worth it, since new viruses, such as the pigeon *Delta*-CoV recently described in China [31], can emerge and spread, and, worldwide, we must anticipate the progression of the viruses with surveillance strategies under a ‘One Health’ scope.

## Data Availability

The data that support the findings of this study are openly available in GenBank at https://www.ncbi.nlm.nih.gov/nucleotide/ (accessed on 3 June 2024) under accession numbers OQ957548-56. The data that support the finding of an inconclusive sequence with many ambiguous positions are available on request from the corresponding authors [J.A.O., A.P.].

## References

[1] Cui J., Li F., Shi Z.L. (2019). Origin and evolution of pathogenic coronaviruses. Nat. Rev. Microbiol..

[2] Adachi S., Koma T., Doi N., Nomaguchi M., Adachi A. (2020). Commentary: Origin and evolution of pathogenic coronaviruses. Front. Immunol..

[3] Hu B., Guo H., Zhou P., Shi Z.L. (2021). Characteristics of SARS-CoV-2 and COVID-19. Nat. Rev. Microbiol..

[4] Miranda C., Silva V., Igrejas G., Poeta P. (2021). Genomic evolution of the human and animal coronavirus diseases. Mol. Biol. Rep..

[5] https://ictv.global/report/chapter/coronaviridae/coronaviridae/orthocoronavirinae.

[6] Nikolaidis M., Papakyriakou A., Chlichlia K., Markoulatos P., Oliver S.G., Amoutzias G.D. (2022). Comparative analysis of SARS-CoV-2 variants of concern, including Omicron, highlights their common and distinctive amino acid substitution patterns, especially at the spike ORF. Viruses.

[7] https://www.who.int/publications/m/item/weekly-epidemiological-update-on-covid-19---1-september-2023.

[8] https://www.ecdc.europa.eu/en/covid-19/variants-concern.

[9] Su S., Wong G., Shi W., Liu J., Lai A.C.K., Zhou J., Liu W., Bi Y., Gao G.F. (2016). Epidemiology, genetic recombination, and pathogenesis of coronaviruses. Trends Microbiol..

[10] Salata C., Calistri A., Parolin C., Palù G. (2019). Coronaviruses: A paradigm of new emerging zoonotic diseases. Pathog. Dis..

[11] Food and Agriculture Organization of the United Nations Animal Health. https://www.fao.org/animal-health/situation-updates/sars-cov-2-in-animals/en.

[12] Cavanagh D. (2005). Coronaviruses in poultry and other birds. Avian Pathol..

[13] Thiermann A.B. (2015). International standards: The World Organisation for Animal Health Terrestrial Animal Health Code. Rev. Sci. Tech..

[14] Toussaint J.F., Sailleau C., Breard E., Zientara S., De Clercq K. (2007). Bluetongue virus detection by two real-time RT-qPCRs targeting two different genomic segments. J. Virol. Methods.

[15] Centers for Disease Control and Prevention (CDC) Coronavirus Disease 2019 (COVID-19), Research Use Only 2019-Novel Coronavirus (2019-nCoV) Real-time RT-PCR Primer and Probe Information. https://www.cdc.gov/coronavirus/2019-ncov/lab/rt-pcr-panel-primer-probes.html.

[16] Corman V.M., Landt O., Kaiser M., Molenkamp R., Meijer A., Chu D.K., Bleicker T., Brünink S., Schneider J., Schmidt M.L. (2020). Detection of 2019 novel coronavirus (2019-nCoV) by real-time RT-PCR. Eurosurveillance.

[17] Ruiz-Arrondo I., Portillo A., Palomar A.M., Santibáñez S., Santibáñez P., Cervera C., Oteo J.A. (2021). Detection of SARS-CoV-2 in pets living with COVID-19 owners diagnosed during the COVID-19 lockdown in Spain: A case of an asymptomatic cat with SARS-CoV-2 in Europe. Transbound. Emerg. Dis..

[18] Hu H., Jung K., Wang Q., Saif L.J., Vlasova A.N. (2018). Development of a one-step RT-PCR assay for detection of pancoronaviruses (α-, β-, γ-, and δ-coronaviruses) using newly designed degenerate primers for porcine and avian `fecal samples. J. Virol. Methods.

[19] Chu D.K., Leung C.Y., Gilbert M., Joyner P.H., Ng E.M., Tse T.M., Guan Y., Peiris J.S., Poon L.L. (2011). Avian coronavirus in wild aquatic birds. J. Virol..

[20] http://www.ncbi.nlm.nih.gov/blast.

[21] Tamura K., Stecher G., Kumar S. (2021). MEGA11: Molecular Evolutionary Genetics Analysis Version 11. Mol. Biol. Evol..

[22] Hepojoki S., Lindh E., Vapalahti O., Huovilainen A. (2017). Prevalence and genetic diversity of coronaviruses in wild birds, Finland. Infect. Ecol. Epidemiol..

[23] Łukaszuk E., Dziewulska D., Stenzel T. (2022). Occurrence and Phylogenetic Analysis of Avian Coronaviruses in Domestic Pigeons (*Columba livia domestica*) in Poland between 2016 and 2020. Pathogens.

[24] Zhuang Q., Liu S., Zhang X., Jiang W., Wang K., Wang S., Peng C., Hou G., Li J., Yu X. (2020). Surveillance and taxonomic analysis of the coronavirus dominant in pigeons in China. Transbound. Emerg. Dis..

[25] Shi J., Wen Z., Zhong G., Yang H., Wang C., Huang B., Liu R., He X., Shuai L., Sun Z. (2020). Susceptibility of ferrets, cats, dogs, and other domesticated animals to SARS-coronavirus 2. Science.

[26] Mastutik G., Rohman A., I’tishom R., Ruiz-Arrondo I., de Blas I. (2022). Experimental and natural infections of severe acute respiratory syndrome-related coronavirus 2 in pets and wild and farm animals. Vet. World.

[27] Lei M., Ma Y., Chen H., Huang P., Sun J., Wang X., Sun Q., Hu Y., Shi J. (2023). Emerging SARS-CoV-2 variants of concern potentially expand host range to chickens: Insights from AXL, NRP1 and ACE2 receptors. Virol. J..

[28] Jonassen C.M., Kofstad T., Larsen I.L., Løvland A., Handeland K., Follestad A., Lillehaug A. (2005). Molecular identification and characterization of novel coronaviruses infecting graylag geese (*Anser anser*), feral pigeons *(Columbia livia*) and mallards (*Anas platyrhynchos*). J. Gen. Virol..

[29] Qian D.H., Zhu G.J., Wu L.Z., Hua G.X. (2006). Isolation and characterization of a coronavirus from pigeons with pancreatitis. Am. J. Vet. Res..

[30] Felippe P.A., da Silva L.H., Santos M.M., Spilki F.R., Arns C.W. (2010). Genetic diversity of avian infectious bronchitis virus isolated from domestic chicken flocks and coronaviruses from feral pigeons in Brazil between 2003 and 2009. Avian Dis..

[31] Wang G.L., Li L.B., Chen J.J., Wang Q.C., Ye R.Z., Li L.M., Zhu K.L., Jiang W.G., Tian S., Fang L.Q. (2023). Emergence of a novel genotype of pigeon Deltacoronavirus closely related to porcine Deltacoronavirus HKU15 and sparrow Deltacoronavirus HKU17 in a live poultry market in Shandong Province, China. Microbiol. Spectr..

